# A Medical Career and Its Prospects

**Published:** 1910-09-03

**Authors:** 


### A MEDICAL CAREER AND ITS PROSPECTS.

BY A GENERAL PRACTITIONER.

The choice of a profession is one of the most momentous steps in a man's life, and when, as is often the case, the decision is made by those in whose care the direction of the youthful life still lies, it should be done with a full sense of the responsibility that such a decision involves and a proper survey of all the conditions and consequences. The first and most important point that naturally comes under consideration is the disposition, inclination, and innate qualities of the young man. The medical profession makes very considerable demands upon the physical and mental resources of the individual. He requires not only, perhaps, something above the average mental capacity, but also a certain robustness of constitution, to enable him to withstand successfully the strain of medical study and hospital life and, later on, the continuous stress involved in an arduous medical career. These considerations alone, therefore, should at once disqualify a certain number of young aspirants to the profession. It may be said of the medical, perhaps more than of any other profession, that either from a thirst for knowledge or for some more indefinable reason a man is impelled towards the profession by very strong feelings, or, on the other hand, everything connected with the magic of healing is distasteful and repellent. He has no desire to know about his own "inside," and strongly dislikes the idea of meddling with other people's. But it may be taken that the larger proportion of youthful candidates about to enter the life struggle for a livelihood through the portals of one of the professions are endowed with a good average intelligence in a healthy body, but displaying no marked abilities, no special bent in any particular direction. Provided, then, that the intelligence be well up to the average, or a little above it, and the body be healthy, the medical profession perhaps offers in return for what it demands as good, and certainly as useful and honourable, a career as a man can obtain in any other direction. But it is not sufficient that a young man should consider merely his tastes and his abilities in relation to the profession, or in relation to the first few years of his student life at the hospital and the examinations he must put successfully behind him; he must also look ahead and determine as far as possible the prospects which he has in view when he has successfully passed through these preliminary stages.

The medical profession covers such a vast field, and includes so many and so varied forms and ways of living and doing, that it would appear to exist rather in the abstract than in the concrete. True, all its members must pass through the same sort of college or hospital training and must be armed with some sort of diploma, but after that the possibilities are so multifarious, the paths of life that may be followed so diverse, that it may be said the medical profession embraces as great a variety of careers as ever can be found outside it. Ordinary general practice alone presents a great variety of conditions according as a practice is chosen for its social life in some fashionable part of town or other centre of society, or in some country district with its rural or sporting pursuits, or rather for the medical work itself in some provincial town with a good local hospital. For the ardent student of science there is the laboratory work at university or hospital, which, though not lucrative, is full of possibilities—possibilities of honour, of satisfaction, and of work accomplished. Then we have the Services—the naval and military, the Colonial and the Indian—each of which offers to certain individuals its peculiar and special attractions, in addition to which there is the consideration that at the end of a certain term of service it is possible to retire with a comfortable, if small, competency and still pursue one's professional activities in some other direction. There are also to be mentioned the lunacy and public health branches of the profession, of which the latter is continually expanding in scope and in its capacity to absorb fresh recruits; and for those of strong religious convictions there is the life of a medical missionary. Finally, for the most ambitious spirits who would strive for the highest places in the profession there are the appointments on the staffs of the universities and hospitals, the stepping-stones to the ranks of medical and surgical consultants and professors.

Now, although it may not be possible or even desirable that a student should map out for himself too definite a course for the future, yet he should, after surveying the various alternatives before him, form some idea for himself of the prospects, conditions, and advantages of these many alternatives, and come to some sort of decision as to which will conform best to his particular tastes and circumstances, rather than enter the profession with no further notion than that of becoming a doctor, so to speak, in the abstract. It is, of course, not in the power of many to say "I will become a member of the staff of a hospital or university." He may be justified in harbouring the laudable ambition of arriving at that distinction, but he must be prepared to accept some humbler path in the profession if circumstances so rule it. On the other hand, a man may enter the profession with some definite idea of joining one of the services, or perhaps of following his father's footsteps in general practice, and may so distinguish himself during his term of studentship that he finds himself appropriated by the staff in due course as one of themselves. In these and in many other ways we find the old proverb, "Man proposes, God disposes," asserting itself. But in spite of these and many other contingencies, it is nevertheless desirable that some sort of preliminary choice should be made in regard to the particular path in the profession which seems most fitting and suitable.

That choice may to some extent influence also the selection of a particular medical school, or determine the particular medical course, examinations, etc., which the student may take.

One word, however, on the question of the medical course and class of examination at which the student aims. It is a great pity, and much to be deplored, when, as not infrequently happens, a student says to himself, "I am only entering for such-and-such a service," or "I am only going into general practice"; "therefore I shall be content with such-and-such a course or examination." That is a bad beginning, and may give cause for serious regrets later on. No matter what sphere of the profession the student may subsequently enter, any knowledge and experience he has acquired will be of service; he cannot equip himself too well either with knowledge and technical skill or with the diplomas which testify to that knowledge. However humble his aspirations may be in regard to his future medical career, let his aims be of the highest in the acquirement of medical knowledge and skill during his days of studentship.

If the student aims high—that is, if he proposes to join the ranks of the consultants—he must be prepared for some hard and unremunerative spade-work during the early years of his professional career. He usually first becomes a demonstrator on the junior teaching staff of the medical school, and he is required to give the best hours of his days in the laboratories, dissecting room, or class rooms with barely sufficient remuneration to provide pocket-money for petty expenses. If he possesses no private means his lot may prove a hard one; but with a position on the junior staff of a medical school there are many little "oddments" to be picked up which together may afford a sufficiency. Even though there may appear no immediate prospect of promotion in his own particular centre, he may always assume the *rôle* of Mr. Micawber, "waiting for something to turn up" with some promise of fulfilment. There are constantly-occurring vacancies of all sorts in all parts of the country and in the Colonies, and it is just from the junior staffs of the medical schools that candidates for these posts are recruited. So long as he remains attached to his hospital or university, in some capacity, no matter how small, he is always "in the swim" and eligible for anything that may come along. Furthermore, it is a fallacy to assume that a small hospital or medical school, with less competition, necessarily offers better chances to those of humbler attainments or abilities, for, be it remembered, the smaller centres are not infrequently staffed with the surplus of able men from the larger institutions. And if the chances of attaining to the position of general surgeon or physician to the hospital be small, in the present age of specialism, there is always the possibility of achieving a considerable reputation among consultants by making a study of some special branch of surgery—gynæcology, aural surgery and rhinology, ophthalmology, or of radiology or bacteriology, all of which offer ample scope for energy and ability, and reasonable prospects of remuneration.

But the mention of bacteriology naturally leads to a consideration of the more scientific side of the profession. For those to whom the more human aspect of a doctor's life—the constant contact with all kinds of humanity involved either in general or consulting practice—does not so strongly appeal, but who find the life of the laboratory more congenial, there is certainly as much if not more scope in the pursuit of medical scientific research than in other spheres of the scientific world. Research work has been sadly neglected in this country in connection with the centres of medical work, but latterly great efforts have been made to amend this deficiency, and there are now constantly being created fresh outlets and new provisions for workers in this most honourable field of labour. Bacteriology affords perhaps the most promise in its scope and extent. For apart from the ordinary bacteriological work at a medical centre, research work and otherwise, the bacteriologist is in ever-increasing demand in connection with all kinds of public health work, the services, and other undertakings; and they are not infrequently accompanied by very comfortable emoluments.

The services have already been referred to. The naval and military services have each their peculiar attractions for those imbued with the naval and military spirit, and the remuneration provided is sufficient for all ordinary needs during the term of service without requiring any further pecuniary assistance from other sources. As already mentioned, there is the further advantage that at the end of a certain term of service, and when a man has still the best years of his life before him, he can retire with a very useful pension and carry on the work of his profession in some other way. Then there is the Colonial service, which offers similar advantages in regard to the pension, and the remuneration is somewhat better, but it is attended with serious danger to health and life owing to the unhealthy climates, such as that of West Africa, to which the medical officers may be consigned. But for those of a good constitution who can well withstand the demands which a hot climate makes upon the health, the Indian medical service offers the best promise. It is a fine service, and in consequence absorbs some of the best of the profession. There is ample scope for work and pleasure, and withal good remuneration, and the possibility of early retirement with a very substantial income. Too much stress cannot be laid upon the fact that one of the chief causes of so much wreckage of human life in the hot climates is the addiction to alcohol, to which so many fall a prey, and one would strongly impress anyone about to enter the Colonial or medical service with the absolute necessity of abstaining altogether from alcoholic beverages, if he desires a successful career and a happy retirement from the service with an unimpaired constitution. He may still have the fever bogey to fight, but abstention will carry him a long way to a successful issue.

The Public Health Department of Medicine provides an ever-increasing number of openings for qualified practitioners, both men and women. They may begin as medical inspectors of schools, with emoluments of £250 to £300 a year, and while occupying these positions may await their opportunity of obtaining a post as full medical officer of health. The work is full of interest and usefulness, and brings the medical man in continual contact with all manner of men and social conditions, and some of the most urgent social reforms may be materially advanced by the energy and ability of the medical officer of health.

Then a word about lunacy. It is a somewhat restricted, limited line of the profession, but perhaps for that matter not more so than other lines of "specialism." Still, it must be borne in mind that it is not only restricted in the scope of the work, but also by the fact that medical men following this line spend a greater part of their existence within the walls of an institution, and lead a life within that institution almost entirely apart from the rest of the community. For those specially interested in psychology and in the physiology and pathology of the brain there is ample scope and opportunity for further investigation and research, and the work should prove sufficiently absorbing. From the pecuniary point of view, the young practitioner obtains board and lodging with a continually increasing "screw," and little outlet for spending it, and the prospect of a house and comfortable income as superintendent of an asylum.

Finally we come to the humble, though honourable and most ancient, calling of the general practitioner. By far the greater number of those who enter the portals of the medical profession eventually, if not immediately, become general practitioners. It is an arduous life, and often a harassing one. The general practitioner is at anyone's beck and call at all hours of the day and night. He is always on duty, and must always be ready for the "call." Even in a slack time, when there may be little regular routine work to do, he still cannot count upon any time of the day as his own, he may be wanted at any moment, and by continual interruptions may be kept on the "go" all day without doing a remunerative day's work. But there are also compensations. The medical practitioner is undoubtedly granted a certain very definite esteem and place of distinction by the rest of the community. Professionally he should be ready for every contingency, and should, therefore, possess the widest range of medical knowledge and skill. Financially, he may have to be content with a very modest income, or his practice may prove a great financial success. Much depends upon circumstances, still more perhaps upon the man. But in making a substantial income of, say, £2,000 or £3,000 a year, his expenses are proportionately large, and, even under the best circumstances, that is to say, in a neighbourhood affording good fees, he must work excessively hard, much harder and for much longer hours than most men are prepared to work in other walks in life. If he possesses some small capital to start with he is saved several years of a toilsome, uphill struggle, and the safest method of investment is probably to start with a partnership in a well-established practice. But before entering upon a permanent practice it is strongly advisable that he should acquire the manner and methods of handling his patients by serving for some time either as a locum tenens or assistant, preferably in some working-class practice in a populous neighbourhood. In this way he may be saved the dangers of having to learn his experience by some lamentable mistakes, which he cannot undo and may find it difficult to live down, once he has started on the practice of his life.

